# Silicon Dioxide Nanoparticles Alter Social Behavior, Color Preference, Oxidative Stress Markers, and Histological Structure of Brain Regions in Zebrafish (*Danio rerio*)

**DOI:** 10.3390/life15111715

**Published:** 2025-11-05

**Authors:** Viorica Rarinca, Irina-Luciana Gurzu, Mircea Nicusor Nicoara, Alin Ciobica, Elena Todirascu-Ciornea, Bogdan Gurzu, Carmen Solcan, Dorel Ureche

**Affiliations:** 1 Doctoral School of Geosciences, Faculty of Geography and Geology, “Alexandru Ioan Cuza” University of Iasi, Carol I Avenue, 20A, 700505 Iasi, Romania; rarinca_viorica@yahoo.com (V.R.); mirmag@uaic.ro (M.N.N.); 2 Doctoral School of Biology, Faculty of Biology, “Alexandru Ioan Cuza” University of Iasi, Carol I Avenue, 20A, 700505 Iasi, Romania; 3 Preclinical Department, “Apollonia” University, Pacurari Street 11, 700511 Iasi, Romania; alin.ciobica@uaic.ro; 4 Department of Preventive Medicine and Interdisciplinarity, Faculty of Medicine, Grigore T. Popa University of Medicine and Pharmacy, 700115 Iasi, Romania; ilgurzu@gmail.com; 5 Department of Biology, Faculty of Biology, “Alexandru Ioan Cuza” University of Iasi, Carol I Avenue, 20A, 700505 Iasi, Romania; ciornea@uaic.ro; 6 Department of Morfofunctional Sciences II, Faculty of Medicine, Grigore T. Popa University of Medicine and Pharmacy, 700115 Iasi, Romania; 7 Preclinical Department, Faculty of Veterinary Medicine, “Ion Ionescu de la Brad” University of Life Sciences of Iasi, 700490 Iasi, Romania; 8 Department of Biology, Faculty of Sciences, “Vasile Alecsandri” University of Bacau, No 157, Marasesti Street, 600115 Bacau, Romania; dureche@ub.ro

**Keywords:** silicon dioxide nanoparticles, immunological parameters, oxidative stress, zebrafish, social interaction test, color preference test

## Abstract

Silicon dioxide nanoparticles (SiO_2_NPs) are widely used in various industries, raising concerns about their potential toxicity in aquatic organisms. Although several studies have investigated the general toxic effects of SiO_2_NPs, little is known about their impact on the nervous system and behavior of aquatic vertebrates. Furthermore, the combined assessment of behavioral, histological, and biochemical responses remains scarce. The study aimed to evaluate the effects of SiO_2_NPs on behavioral, histological, and biochemical parameters in adult zebrafish (*Danio rerio*). Fish were exposed to sublethal concentrations of SiO_2_NPs and their behavior was assessed using the social interaction test and the color preference test. Significant alterations in social behavior, such as reduced group cohesion and increased isolation tendencies, were observed. Additionally, exposed zebrafish exhibited a marked shift in color preference, indicating potential disruptions in sensory or cognitive function. Histological analyses revealed dose dependent tissue changes in brain structures, while biochemical assays indicated reduced activity of antioxidant enzymes, including catalase (CAT), superoxide dismutase (SOD), and glutathione peroxidase (GPx), suggesting elevated oxidative stress (OS). To the best of our knowledge, this is one of the first studies to integrate behavioral, histological, and biochemical endpoints in zebrafish to assess the neurotoxic potential of SiO_2_NPs. These findings suggest that SiO_2_NPs can induce histological alterations in brain structures, neurobehavioral changes, and OS in zebrafish, underscoring the novelty and relevance of this interdisciplinary approach, and the importance of further studies on SiO_2_NPs environmental and health impacts.

## 1. Introduction

Nanotechnology is one of the fastest growing and most promising scientific fields, with applications ranging from medicine, electronics, catalysis, cosmetics to targeted drug delivery [1]. As engineered nanomaterials, nanoparticles offer unique physicochemical properties: their nanoscale dimensions and large surface-to-volume ratio enable them to establish close interactions with biological systems [2]. On the other hand, their increased use raises serious concerns about potential human health and environmental risks throughout their entire life cycle from production and use to disposal and release into the environment [3].

SiO_2_NPs are among the most used nanomaterials due to their stability, biocompatibility, and anti-agglomeration properties [4]. These particles are commonly found in various consumer products such as pharmaceuticals, cosmetics, food additives and inks, with increasing applications in biomedical fields, particularly in drug and gene transport and cancer therapies [5].

Beyond these uses, silica nanoparticles also have wide industrial applications, including in the production of paints, coatings, plastics, concrete, ceramics, and as additives in the electronics and energy industries [6].

Due to their extensive industrial use and poor removal efficiency in wastewater treatment plants, SiO_2_NPs can be released into aquatic ecosystems through industrial effluents, surface runoff, and sewage discharge, ultimately accumulating in freshwater environments [7].

Environmental monitoring and modeling studies have estimated that the predicted environmental concentrations (PECs) of silica nanoparticles in surface waters range between 0.12 and 2.6 μg L^−1^ [8,9], depending on proximity to industrial and wastewater discharge sources. These values emphasize the environmental relevance of assessing the biological effects of SiO_2_NPs in aquatic organisms such as zebrafish.

At the same time, due to their ability to penetrate cells and accumulate in tissues, there is a growing need to evaluate their biocompatibility, biodistribution and potential toxicity [10].

Traditional in vivo toxicity testing using rodents, although informative, faces ethical limitations, high costs and time constraints [11]. As an alternative, the zebrafish model has gained importance in nanotoxicology due to its genetic similarity to humans, transparency during early development and suitability for high-throughput screening [12]. Notably, the use of zebrafish allows for an integrated assessment of behavioral, histological, and biochemical effects of nanomaterials, which remains underexplored for SiO_2_NPs. Transgenic zebrafish lines expressing tissue-specific fluorescent proteins allow real-time visualization of the developmental and vascular dysfunctions induced by nanomaterials [13].

The present study investigated the in vivo behavioral effects of SiO_2_NPs using the sociability test and the color preference test along with histological and biochemical analyses. This integrated approach is among the first to combine neurobehavioral, structural, and biochemical endpoints in zebrafish exposed to SiO_2_NPs, highlighting the novelty and interdisciplinary relevance of our work. The aim was to investigate how these nanoparticles influence the social behavior and sensory preferences of zebrafish, as well as possible structural and biochemical changes at the organ level, with a focus on biomarkers of OS.

## 2. Materials and Methods

### 2.1. Zebrafish

The experiment used adult zebrafish (AB strain), aged between 6 and 8 months at an equal male-female ratio (1:1). The fish were obtained from an authorized local breeder and acclimatized for seven days in tanks under controlled laboratory conditions (14:10 light/dark cycle, pH 7.2 and constant 28 °C) and were fed twice daily with TetraMin flakes [14].

The experiments performed on zebrafish were in accordance with the following: EU Commission Recommendation (2007) and Directive 2010/63/EU of the European Parliament and of the Council of 22 September 2010 on the protection of animals used for scientific purposes [15]; Declaration of Helsinki; the legislation of Romania and of the European Union regarding the use of animals in biomedical research. The experiments and procedures used were approved by the Committee of Ethics of the Faculty of Biology (“Alexandru Ioan Cuza” University of Iasi), the Approval Code being no. 100/10.12.2024. The ARRIVE guidelines were also kept in mind by limiting the number of zebrafish used [16]. After the exposure period and completion of behavioral assessment, zebrafish were euthanized by placing them in ice-cold water for 10 min. This method is recommended to minimize discomfort in small fish [17].

### 2.2. Materials

Silica nanoparticles (SiO_2_NPs, CAS number 7631-86-9, particle size 50 nm) were purchased from Sigma-Aldrich (Saint Louis, MO, USA). SiO_2_NP suspensions were prepared by adding the appropriate amount of dry nanoparticles directly to system water in 5 L glass aquaria. Suspensions were sonicated for 30 min at room temperature to promote uniform dispersion. Concentrations of 100 and 500 μg mL^−1^ were selected based on previous toxicological studies that demonstrated neurobehavioral and oxidative effects in zebrafish at similar or higher exposure levels and to allow investigation of potential dose-dependent responses under controlled exposure conditions [18,19]. Although characterization of nanoparticles in suspension (e.g., DLS) was performed in this study, the dispersion protocol followed commonly applied mild sonication conditions that are not expected to alter nanoparticle physicochemical properties of nanoparticles.

### 2.3. Zebrafish Exposed to SiO_2_NPs

Adult zebrafish were divided into three experimental groups: the control group and two groups exposed to SiO_2_NPs at concentrations of 100 μg mL^−1^ (group 2) and 500 μg mL^−1^ (group 3), respectively, under controlled laboratory conditions. Fish were exposed in 5 L glass aquaria, each containing ten adult zebrafish (1:1 male-to-female ratio), corresponding to approximately one fish per 500 mL of water, in accordance with OECD guidelines for zebrafish testing. After exposure, behavioral tests (social interaction test, color preference test) were performed and OS markers (CAT, SOD, GPx) and immunological parameters (glial fibrillary acidic protein (GFAP), proliferating cell nuclear antigen (PCNA), calcium binding protein S100 β (S100β)) were examined to evaluate the potential neurotoxic and physiological effects induced by SiO_2_ nanoparticles.

### 2.4. Experimental Setup for Behavioral Testing

Following a 7-day exposure to SiO_2_NPs, zebrafish were investigated for social interactions and color preferences using a T-maze. The setup included three distinct arms: central, left, and right. To monitor and record the fish’s activity, a camera is placed above the behavioral apparatus. To assess color preference, the left and right arms of the maze were visually modified: one arm was colored red and the other green. These colors were selected based on previous studies of zebrafish behavior, as zebrafish possess trichromatic vision and can reliably distinguish between red and green stimuli [20]. In the social interaction test, a conspecific was introduced in the left arm, behind a transparent partition, while the opposite arm was left empty. This design allowed for the quantification of social behavior based on the time spent by the tested fish in each arm. Spending more time in the arm with the social stimulus indicated stronger social behavior. In contrast, spending more time in the right arm, while avoiding the other arm, was interpreted as anxiety-related behavior.

### 2.5. Acclimation for Color Preference Test

The color preference test was performed in a T-maze. The experimental protocol involved a structured acclimation phase, followed by a detailed assessment of behavioral indicators related to locomotion, anxiety-like responses, and color preferences. To obtain consistent and reliable data, zebrafish were gradually acclimated to the test environment over several days. On the first day, the entire group *(n* = 12) of adult zebrafish was introduced into the maze for a period of 20 min. On the following days, both the group size and the duration of exposure were progressively reduced—half of the group (*n* = 6) for 10 min, 3–4 individuals for 8 min, 2–3 individuals for 5 min, and finally 1–2 zebrafish for 5 min. On the last day of acclimation, individual fish were introduced for 5 min of exploration. The effectiveness of acclimation was assessed based on swimming patterns; specimens that exhibited frequent immobility or lack of exploratory movement were excluded from further behavioral analysis [21].

The behavioral parameters investigated for color preference consisted of total distance traveled (cm), swimming velocity (cm s^−1^), time spent in the right and left arms of the T-maze during the 5 min test, cumulative duration of mobility (s) and cumulative duration of immobility (freezing behavior) (s), and rotational behavior measured in both clockwise and counterclockwise directions.

The behavioral parameters investigated for the social interaction test consisted of total distance traveled (cm), swimming velocity (cm s^−1^), and time spent in the left and right arms of the test T-maze. The left arm contained a transparent compartment housing conspecifics (stimulus arm), while the right arm remained empty (neutral arm).

### 2.6. Preparation of Homogenates and Biochemical Parameter Analysis

After completion of behavioral tests, zebrafish were immersed in a glass container with ice-cold water for 10 min, followed by rapid euthanasia, according to the method reported by Jorge et al. [22]. The next day, fish from the same group were individually weighed and homogenized in potassium phosphate extraction buffer (0.1 M potassium phosphate buffer, pH 7.4, containing 1.15% potassium chloride) at a ratio of 1:10 (weight/volume). The homogenates were then centrifuged at 3500 rpm for 15 min, and the resulting supernatant was used for subsequent analyses of biochemical parameters (*n* = 3 for each group).

#### 2.6.1. Determination of Superoxide Dismutase Activity

SOD activity was assessed using an adapted method described by Winterbourn et al. [23]. This approach is based on the ability of the enzyme to prevent the reduction of nitroblue tetrazolium (NBT) by superoxide anions generated by the photoreduction of riboflavin. The reaction mixture consisted of 0.067 M potassium phosphate buffer (pH 7.8), the enzyme extract, 0.1 M ethylenediaminetetraacetic acid (EDTA) serving as a chelating agent, 0.12 mM riboflavin as a source of superoxide radicals, and a 1.5 mM NBT solution as an electron acceptor. The reaction was initiated by exposure to light and the inhibition of NBT reduction was monitored spectrophotometrically at 560 nm. Enzyme activity was adjusted according to protein concentration and expressed in units per milligram of protein [24]. One unit of SOD activity was defined as the amount of enzyme required to inhibit NBT reduction by 50% under the assay conditions.

#### 2.6.2. Determination of Catalase Activity

CAT activity was assessed using the colorimetric assay reported by Sinha [25]. This assay is based on the quantification of hydrogen peroxide (H_2_O_2_) remaining after enzymatic decomposition. Each sample consisted of 125 µL of tissue homogenate mixed with 125 µL of 0.16 M H_2_O_2_ solution. The reaction mixture was allowed to incubate at room temperature for 3 min to allow the reaction to proceed. To terminate the enzymatic reaction, 500 µL of a chromogenic reagent composed of potassium dichromate in glacial acetic acid was added. This reagent reacts with any residual H_2_O_2_ to form a stable-colored complex. The mixture was then heated at 95 °C for 10 min to develop the color. After cooling, the samples were centrifuged at 14,000 rpm for 5 min to separate the supernatant, and the absorbance was recorded at 570 nm using a spectrophotometer. CAT activity was quantified by the rate of H_2_O_2_ decomposition (μmol min^−1^) and standardized to protein concentration (mg).

#### 2.6.3. Determination of Glutathione Peroxidase Activity

GPx activity was assessed by a spectrophotometric assay based on the method of Fukuzawa and Tokumura [26]. This assay evaluates the ability of the enzyme to catalyze the reduction of H_2_O_2_ using reduced glutathione (GSH) as an electron donor, resulting in the conversion of GSH to its oxidized form (G-S-S-G) and the formation of water. Each assay consisted of 78 µL of enzyme extract combined with 475 µL of 0.25 M sodium phosphate buffer (pH 7.4), 36 µL of 25 mM EDTA, and 36 µL of 0.4 M sodium azide. The reaction mixture was maintained at 37 °C for 10 min to ensure that the reaction reached a steady state. To initiate the enzyme-catalyzed reaction, 50 µL of 50 mM GSH and 36 µL of 50 mM H_2_O_2_ were added after incubation. The addition of 730 µL of 7% metaphosphoric acid stopped the reaction, and the samples were centrifuged at 14,000 rpm for 10 min to remove precipitates. Then, 100 µL of the supernatant was taken and combined with 1270 µL of 0.3 M disodium phosphate buffer and 136 µL of 0.04% 5,5′-dithiobis-(2-nitrobenzoic acid) (DTNB) solution. Absorbance at 412 nm was measured after 10 min of incubation, and GPx activity was expressed in enzyme units per milligram of protein. One unit of GPx activity was defined as the amount of enzyme that catalyzes the oxidation of 1 μmol of GSH per minute under the assay conditions.

### 2.7. Histological and Immunohistological Procedures

Organ samples were fixed in Bouin’s solution for 24 h. Sections of approximately 0.5 cm thickness were dehydrated with a decreasing concentration of ethanol, cleared in xylene, and embedded in paraffin. Tissue sections were cut at a thickness of 5 µm for hematoxylin and eosin staining and at a thickness of 4 µm for immunohistochemistry (IHC) using a rotary microtome. After sectioning with a microtome, 10 microscope slides were selected from each paraffin block, specifically stained, and read on a CX41 microscope (Olympus, Hamburg, Germany) (*n* = 3 fish per group).

For IHC, sections were deparaffinized in xylene, rehydrated in ethanol, and subjected to heat-induced antigen retrieval by heating in a microwave for 20 min at medium-high power, with the temperature monitored in a separate container filled with citrate buffer (10 mmol, pH 6) maintained at 95 °C. After cooling for 20 min, the slides were washed twice in phosphate-buffered saline (PBS), for 5 min each. Sections were treated with 3% H_2_O_2_ to block endogenous peroxidase activity, then rinsed in PBS.

Primary antibodies were applied overnight at 4 °C in a humidified atmosphere as follows: rabbit polyclonal anti-GFAP antibody (1:1000 dilution, cat. no. 173002, Synaptic Systems, Goettingen, Germany), rabbit monoclonal anti-S100β antibody (1:1000 dilution, cat. no. C48942, Signalway Antibody), and rabbit polyclonal anti-PCNA antibody (1:250 dilution, cat. no. PA5-27214, Thermo Fisher Scientific, Waltham, MA, USA). The following day, slides were washed three times in PBS (5 min each) and incubated with the NovoLink™ Polymer Detection System (goat anti-rabbit IgG secondary antibody; cat. no. RE7140-K, Leica Biosystems). Immunoreactivity was visualized using 3,3′-diaminobenzidine (DAB) as a chromogen and finally counterstained with hematoxylin. All histological and immunohistochemical images were prepared at a minimum resolution of 600 dpi and quantitatively analyzed using QuPath software.

### 2.8. Statistical Analysis

The normality of the distribution of the dataset was assessed using the Shapiro–Wilk test, implemented in GraphPad Prism 9 (GraphPad Software, San Diego, CA, USA). Outliers were detected by the Grubbs test and removed as necessary. Based on the normality results, statistical analysis was performed using one-way analysis of variance (ANOVA), followed by Tukey’s post hoc test for pairwise comparisons. Data are expressed as mean ± standard deviation (SD), and statistical significance was considered at *p* values less than 0.05.

## 3. Results

### 3.1. Social Interaction Test

**Figure 1:**
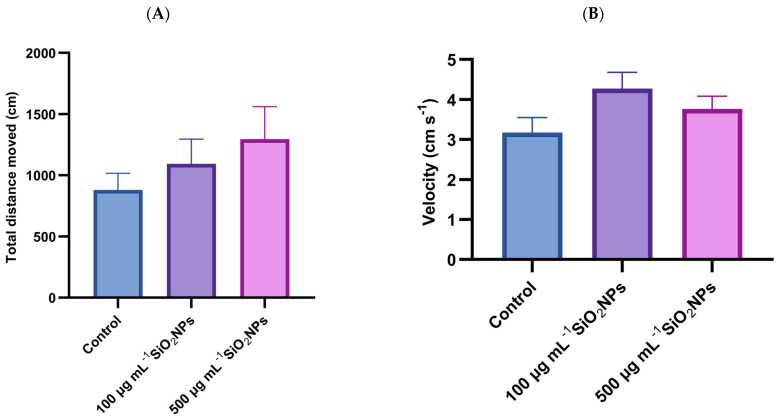
Effects of exposure to SiO_2_NPs on locomotor activity during the social interaction test in zebrafish. (**A**) Total distance traveled (cm); (**B**) Velocity (cm s^−1^). Data are expressed as mean ± SD (*n* = 10 per group). No statistical analysis was performed for this dataset.

The results of the social interaction test show a tendency for locomotor activity to increase with increasing concentration of SiO_2_NPs (Figure 1). The groups that were administered SiO_2_NPs had increased values compared to the control group, but there were no significant differences, with the greatest distance traveled being achieved by the experimental group that was administered 500 μg mL^−1^.

This pattern could suggest that SiO_2_NPs stimulate locomotor activity, potentially through neurochemical changes, including increased levels of dopamine or serotonin, which are linked to increased exploratory behavior. At the same time, the increase in movement could also be interpreted as a possible response to stress or agitation, caused by exposure to nanomaterials. Also, in the case of velocity, visible but not significant differences are observed, with higher values observed in the groups that were administered SiO_2_NPs compared to the control group. In conclusion, although statistical significance is not demonstrated, the data suggest that exposure to SiO_2_NPs could influence the locomotor behavior of zebrafish, with a possible dose-dependent effect.

**Figure 2:**
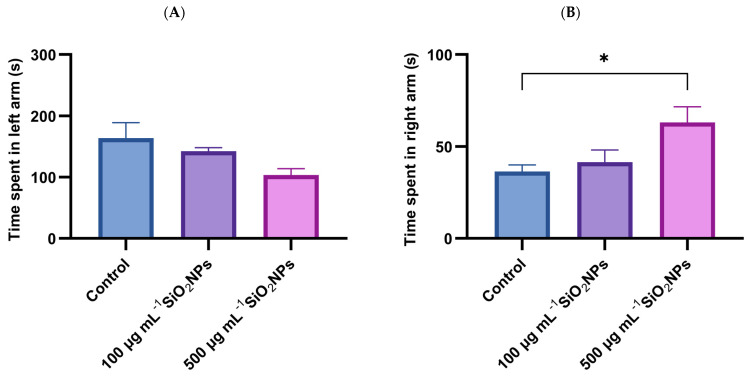
Effects of exposure to SiO_2_NPs on social behavior in zebrafish. (**A**) time spent in left arm (s). (**B**) Time spent in right arm (s). Data are expressed as mean ± SD (*n* = 10 per group). No statistical analysis was performed for this dataset. Asterisks indicate statistical significance: * *p* < 0.05.

In addition to velocity and total distance traveled, two other specific parameters were analyzed, namely the time spent in the stimulus arm (left arm) and the time spent in the non-stimulus arm (right arm) (Figure 2).

The results show that exposure to SiO_2_NPs affects the social behavior of zebrafish, evidenced by the reduction in the time spent in the left arm, correlated with the increase in the concentration of SiO_2_NPs, as well as a concomitant increase in the time spent in the non-stimulus arm.

### 3.2. Color Preference Test

**Figure 3:**
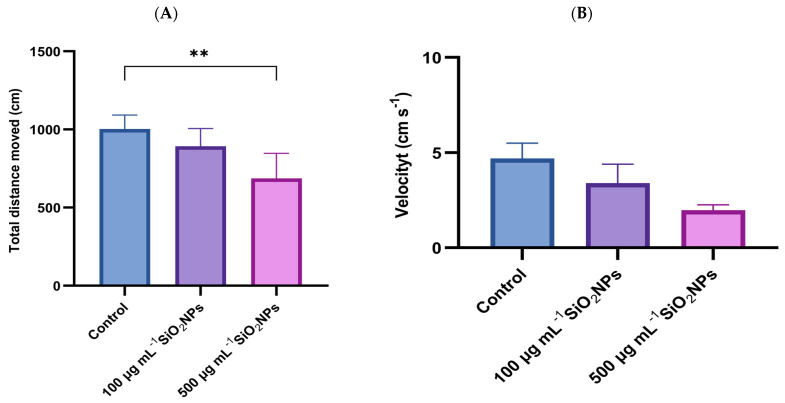
Effects of exposure to different concentrations of SiO_2_NPs on locomotor activity during the color preference test in zebrafish. (**A**) Total distance traveled (cm); (**B**) Velocity (cm s^−1^). Data are expressed as mean ± SD (*n* = 10 per group). Statistical analysis was performed using one-way ANOVA followed by Tukey’s post hoc test. Asterisks indicate statistical significance: ** *p* < 0.01.

Color preference was assessed through a specific test, which measured velocity and total distance traveled (Figure 3).

Regarding total distance traveled, a progressive decrease in this parameter was observed with increasing concentration of SiO_2_NPs, the difference becoming statistically significant between the control group and the one exposed to the highest concentration (*p* = 0.004).

Similarly, velocity tended to decrease with higher concentrations of SiO_2_NPs, indicating changes in locomotor behavior, possibly related to reduced activity or inhibitory responses triggered by nanoparticle exposure.

**Figure 4:**
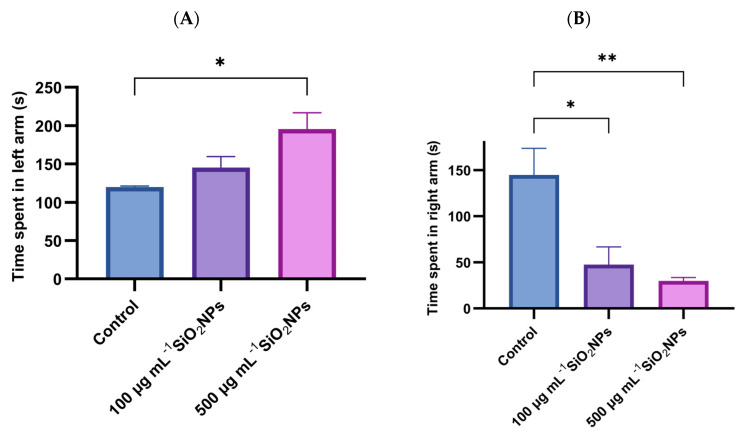
Effects of exposure to SiO_2_NPs on zebrafish behavior. (**A**) Total time (s) spent in the left arm. (**B**) Total time (s) spent in the right arm. Data are expressed as mean ± SD (*n* = 10 per group). Statistical analysis was performed using one-way ANOVA followed by Tukey’s post hoc test. Asterisks indicate statistical significance: * *p* < 0.05; ** *p* < 0.01.

The time spent in the right arm (green) and the time spent in the left arm (red) in the color preference test were also analyzed (Figure 4). The results indicated a significant increase in the time spent in the left arm with increasing concentration of SiO_2_NPs, the difference becoming significant between the control group and the one that was administered SiO_2_NPs with a higher concentration (*p* = 0.027). In parallel, the time spent in the right arm (green) recorded a significant decrease in both treated groups, compared to the control group: 100 μg mL^−1^ (*p* = 0.019) and 500 μg mL^−1^ (*p* = 0.008). These changes suggest an influence of SiO_2_NPs on exploratory behavior and chromatic preference.

**Figure 5:**
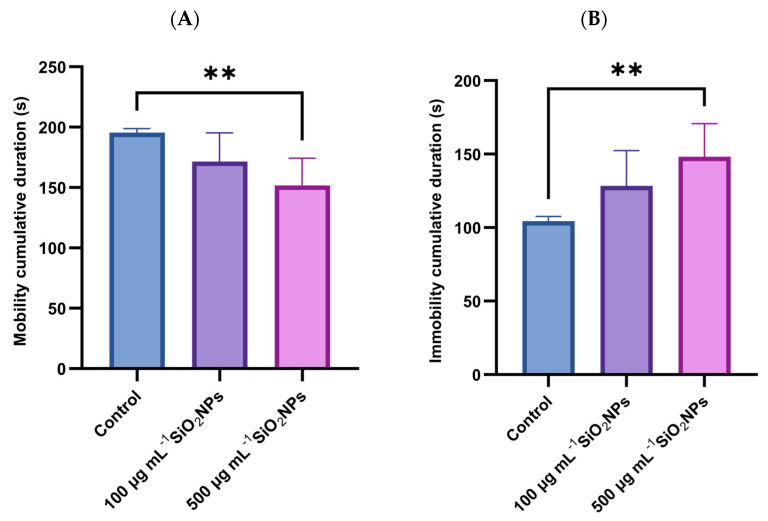
Effects of exposure to SiO_2_NPs on social behavior in zebrafish. (**A**) Cumulative duration of mobility (s). (**B**) Cumulative duration of immobility (s). Data are expressed as mean ± SD (*n* = 10 per group). Statistical analysis was performed using one-way ANOVA followed by Tukey’s post hoc test. Asterisks indicate statistical significance: ** *p* < 0.01.

Two additional behavioral parameters examined in the experiment were the cumulative duration of mobility and the cumulative duration of immobility (Figure 5). The results revealed a progressive decrease in mobility and an increase in immobility depending on the concentration of SiO_2_NPs administered. Thus, the duration of mobility was significantly reduced in the group that was administered the higher concentration of SiO_2_NPs compared to the control group (*p* = 0.0088). Correspondingly, the duration of immobility showed a significant increase at the same concentration, compared to the control group (*p* = 0.0088), suggesting a possible inhibitory effect on spontaneous motor activity, associated with exposure to high doses of SiO_2_NPs.

**Figure 6:**
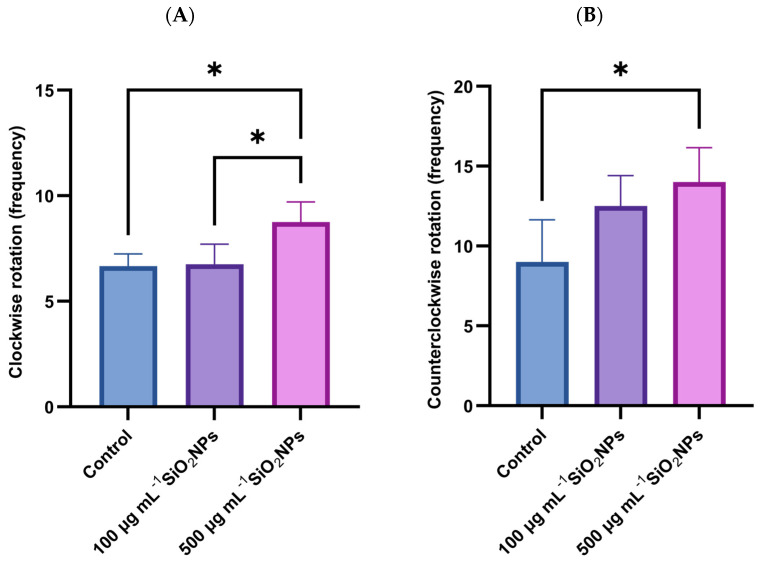
Effects of exposure to SiO_2_NPs on social behavior in zebrafish. (**A**) Clockwise rotation (frequency). (**B**) Counterclockwise rotation (frequency). Data are expressed as mean ± SD (*n* = 10 per group). Statistical analysis was performed using one-way ANOVA followed by Tukey’s post hoctest. Asterisks indicate statistical significance: * *p* < 0.05.

In the color preference test, a significant increase in the frequency of clockwise rotations was observed in individuals exposed to the higher concentration of SiO_2_NPs (Figure 6), compared to both the control group (*p* = 0.034) and the group exposed to the lower concentration (*p* = 0.0294). Also, in the case of counterclockwise rotations, a significant increase was recorded in the group treated with the higher dose compared to the control group (*p* = 0.0427), which may indicate a disruption of exploratory behavior or sensory response, possibly associated with the neurotoxicity induced by SiO_2_NP.

### 3.3. Oxidative Stress

**Figure 7:**
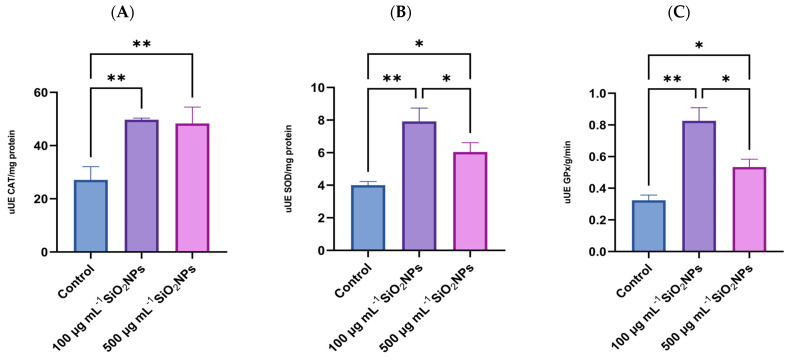
Effects of exposure to SiO_2_NPs on biomarkers of oxidative stress in zebrafish: (**A**) Catalase (CAT); (**B**) Superoxide Dismutase (SOD); (**C**) Glutathione Peroxidase (GPx). Data are expressed as mean ± SD (*n* = 3 per group). Statistical analysis was performed using one-way ANOVA followed by Tukey’s post hoc test. Asterisks indicate statistical significance: * *p* < 0.05; ** *p* < 0.01.

Regarding OS biomarkers, three essential antioxidant enzymes in cellular defense against reactive oxygen species (ROS) were analyzed: SOD, GPx, and CAT (Figure 7). These enzymes play a central role in maintaining redox balance, contributing to the neutralization of free radicals and the prevention of lipid peroxidation. Their activity was evaluated to highlight the changes induced by the administration of SiO_2_NPs and to characterize the adaptive antioxidant response of the organism, depending on the applied concentration.

Regarding CAT activity, a statistically significant increase was observed between the control group and the group that was administered the lower concentration of SiO_2_NPs (*p* = 0.0098), as well as between the control group and the one treated with 500 μg mL^−1^ SiO_2_NPs, in terms of antioxidant activity measured after exposure.

SOD activity also increased in both treated groups compared to the control group, with a more pronounced increase in the lower concentration compared to the control group (*p* = 0.0027). At the same time, a significant decrease in SOD activity was observed in the group that received the higher concentration of SiO_2_NPs compared to the one treated with 100 μg mL^−1^ (*p* = 0.0494), which may indicate a possible inhibitory or depletion effect of the antioxidant response at higher doses.

Regarding GPx activity, enzyme levels were significantly higher in the 100 μg mL^−1^ treated group compared to the control group (*p* = 0.0011), as well as compared to the group that was administered the higher concentration of nanoparticles (*p* = 0.0122). Similarly, in the group exposed to the higher concentration of nanoparticles, GPx activity was also significantly increased compared to the control group (*p* = 0.0277), suggesting a potential redox imbalance or impairment of antioxidant capacity.

### 3.4. Histological and Immunohistological Results

**Figure 8:**
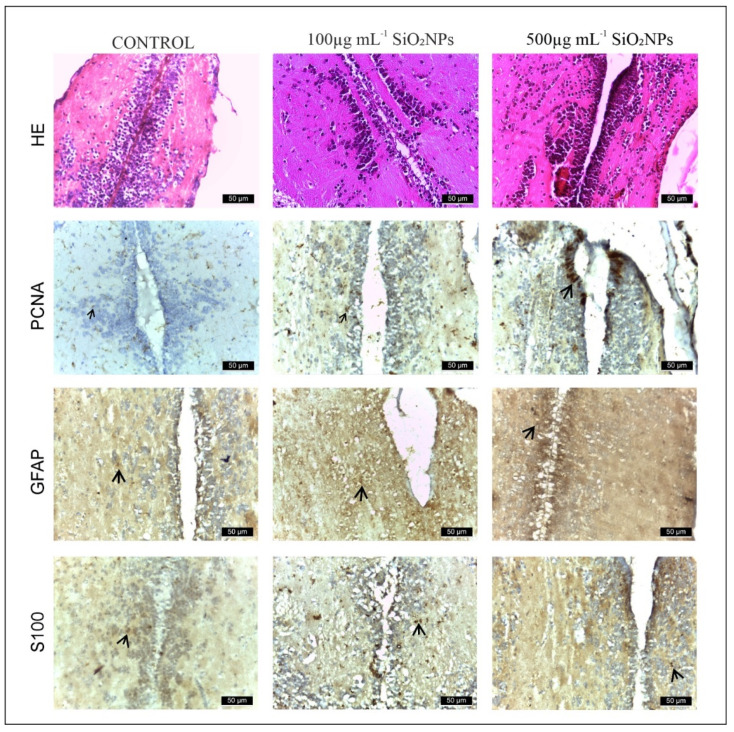
Appearance of the rostro-caudal axis of the telencephalon in zebrafish from control and experimental groups: HE, PCNA, GFAP and S100 stain. Scale bar: 50 μm. Arrowheads indicate immunopositive cells.

This experiment investigated the neurotoxic effect of SiO_2_NPs. The study focused on the telencephalon and mesencephalon. PCNA, GFAP, and S100β antibodies were used. Exposure to SiO_2_NPs produced neurotoxic effects in adult zebrafish characterized by neuropil degeneration in zone III of the white matter of the optic tectum, areas of neuronal necrosis in the perigranular zone of the optic tectum, activation and proliferation of radial and ependymal glial cells that showed numerous GFAP-labeled extensions in TeV. We believe that SiO_2_ NPs also activated areas of regeneration in both the telencephalon (Figure 8) and optic tectum (Figure 8, as indicated by the labeling of these cells with PCNA.

**Figure 9:**
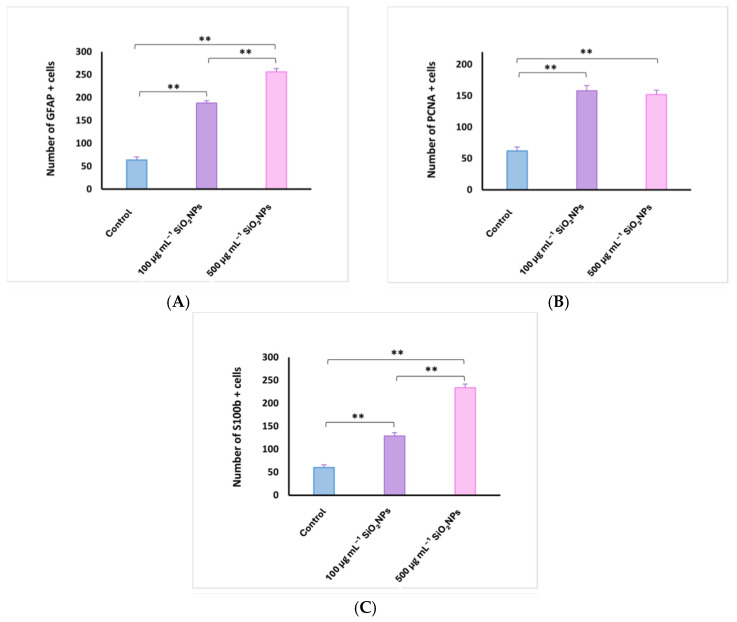
Quantification of glial fibrillary acidic protein (GFAP) (**A**), proliferating cell nuclear antigen (PCNA) (**B**), and S100 calcium-binding protein B (S100B) (**C**) cells in the telencephalon of adult zebrafish after exposure to SiO_2_NPs. Data are expressed as mean ± SEM. ** *p* < 0.01.

Areas of the telencephalon in the control and experimental groups stained with GFAP, PCNA and S100β IHC. In the control group, glial cells labeled with GFAP (64 ± 14.87, Figure 9) and S100-labeled glial cells (60 ± 13.66) were located mainly in the telencephalic ventricle (TelV). PCNA cells are reduced in number in the control group (62 ± 13.93). The group exposed to 100 μg mL^−1^ SiO_2_NPs is distinguished by a very significant increase in the number of cells labeled with the three antibodies GFAP (188 ± 11.70), PCNA (158 ± 18.56) and S100 (129 ± 15.18), respectively (Figure 8), especially in the central and lateral nuclei of the dorsal and ventral telencephalic zone, but also by a reduction in the number of neurons in these areas. The group exposed to 500 μg mL^−1^ SiO_2_NPs also showed a highly significant increase in the number of GFAP (256 ± 17.26), S100 (234 ± 17.10) and PCNA-labeled cells (152 ± 15.52) in the telencephalic ventricle area. Ependymal cells lining the telencephalic ventricle in the rostral area were PCNA-positive in greater numbers compared to the other groups. Many positively labeled cells were also observed in the dorsal and ventral telencephalic area. In addition, a reduction in neurons and vessel ectasia, as well as their congestion, was observed in the lateral areas of the telencephalic.

**Figure 10:**
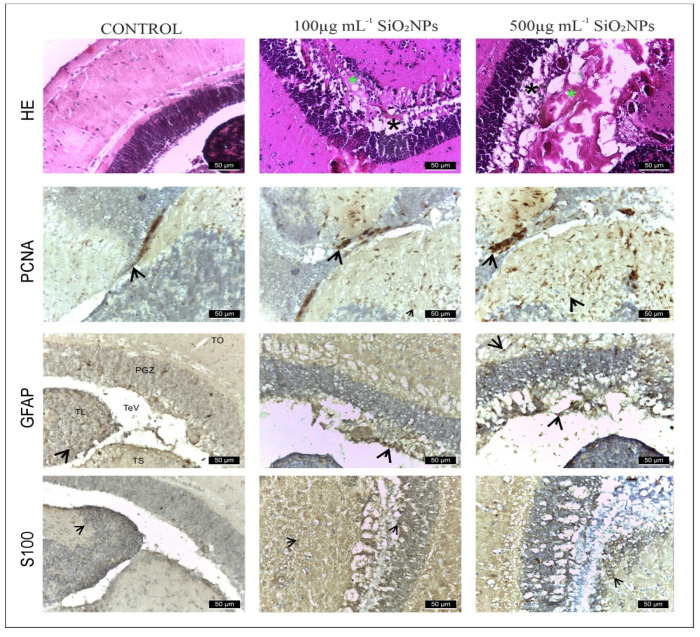
Histology of adult zebrafish brain from control and experimental groups. The untreated adult zebrafish brain shows intact structural morphology and cellular morphology. The 4 layers of the optic tectum: (I) superficial gray-white zone, (II) central zone, (III) deep white zone, and (IV) periventricular gray zone (PGZ). The lateral torus (TLa) is homogenous, diffusively arranged, with no apparent divisions or inflammation. The brain section has undamaged cellular morphology which represents non-existent inflammation. Rows show IHC staining for anti-GFAP, anti-PCNA, and anti-S100. First row shows hematoxylin-eosin (HE) staining. Scale bar: 50 μm. Rows show IHC staining for anti-GFAP, anti-PCNA and anti-S100. First row shows hematoxylin-eosin (HE) staining. Scale bar: 50 μm. Arrowheads indicate immunopositive cells. Symbols: *—edema area (green); *—neuronal rarefaction (black).

The midbrain comprises the tectum opticum (TeO) (Figure 10), bordered medially by the torus longitudinalis of radial glial cells (RG), as described in TeO, with the bodies of these cells aligned along the deepest tectal layer of the ventricle (periventricular gray area) and extending a basal process that reaches the upper layers of TeO. The terminal extensions of these RG cells form a glial boundary in the more superficial marginal layers and come into contact with blood vessels [26]. However, this arrangement of the RG excludes the marginal zone of proliferation of TeO, where RG cells are not present. Tectal RG cells express markers such as GFAP and S100β [27,28]. RG-expressing markers are also observed along the tegmental ventricle and the torus semicircularis, although their processes are shorter and/or do not extend radially [29].

In the control group (Figure 10), GFAP and S100 antibodies labeled a reduced number of glial cells (121 ± 19.04 and, respectively, 115 ± 17.10) (Figure 10, Table S1), observed in both the white matter of the caudal tectal commissure (Ctec) and in the gray matter of the optic tectum, torus semicircularis, and torus lateralis. PCNA labeled a small number of cells in the ventromedial area, also known as the posterior mesencephalic lamina (PML) (56 ± 3.54). In the HE column, the granular layer of neurons in the PGZ area is compact and appears normal.

The group exposed to 100 mg L^−1^ SiO_2_NPs showed a proliferation of GFAP-labeled glial cells (146 ± 18.11), but not statistically significant, but the number of S100-labeled cells increased significantly in the PGZ layer (154 ± 17.68) (Figure 10), with long extensions in the TeV and small areas of neuronal necrosis in the PZG layer.

PCNA-labeled cells, involved in neuroregeneration during the DNA synthesis phase, are present in the peripheral zone of the diencephalic ventricle (Div), the ventromedial optic tract, and have increased significantly (153 ± 7.91) (Figure 10). Small areas of neuropil degeneration were observed in the white matter in the external vicinity of the PGZ layer. Blood vessel ectasia also appeared by HE staining in TeV.

**Figure 11:**
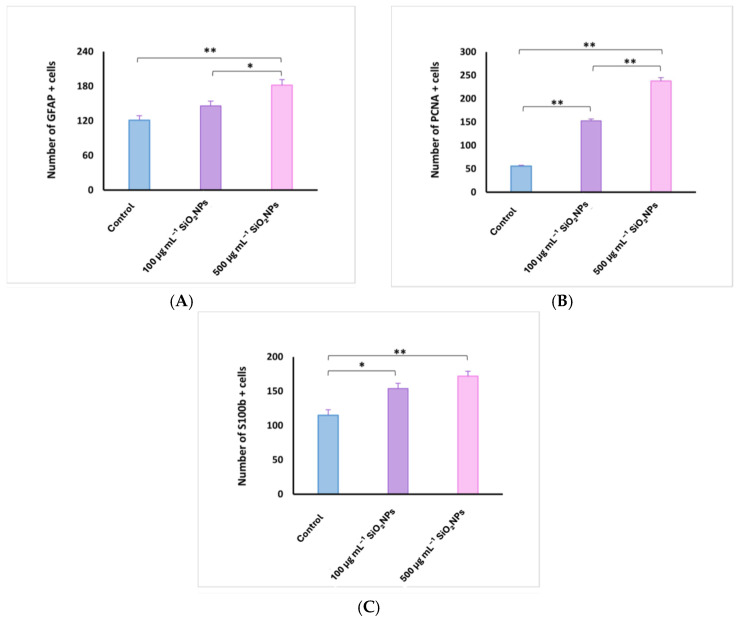
Quantification of glial fibrillary acidic protein (GFAP) (**A**), proliferating cell nuclear antigen (PCNA) (**B**), and S100 calcium-binding protein B (S100B) (**C**) cells in the optic tectum of adult zebrafish after exposure to SiO_2_NPs. Data are expressed as mean ± SEM. * *p* < 0.05; ** *p* < 0.01.

Optic tectum lesions in fish exposed to 500 μg mL^−1^ SiO_2_NPs are more pronounced compared to the group exposed to 100 μg mL^−1^. The changes are represented by a very significant proliferation of GFAP (182 ± 21.55) and S100 (172 ± 15.81) labeled glial cells evident in the PGZ layer with long extensions and TeV (Figure 11), several areas of neuronal necrosis in the PGZ layer and a reduction in the diameter of this layer.

The internal zone located on the inner surface of the optic tectum in the tectal ventricle was lined with S100-positive cells that exhibit morphological characteristics of ependymal and subependymal cells. PCNA-labeled cells were also very significantly increased (238 ± 15.51), being more numerous in the torus semicircularis (TSc), periventricular diencephalon (Div), and posterior mesencephalic lamina (LMP). In the white matter of the third zone of the optic tectum, frequent small areas of neuropil degeneration were observed. HE staining also shows ectasia and congestion of blood vessels in the TeV, as well as a slightly reduced diameter of the PGZ granular layer.

Taken together, these findings suggest that SiO_2_NPs exert neurotoxic effects in adult zebrafish by inducing OS, triggering glial activation (S100β, GFAP), promoting apoptosis, and causing structural alterations in the telencephalon and optic tectum, which together contribute to the observed behavioral changes.

## 4. Discussion

The present study investigated the behavioral, biochemical, and histological effects of SiO_2_NPs on adult zebrafish, focusing on social interaction, color preference, locomotor activity, OS biomarkers, and histopathology. The results indicate a complex, concentration-dependent impact of SiO_2_NPs exposure on both behavioral phenotypes and antioxidant defense mechanisms.

The concentrations of 100 and 500 μg mL^−1^ were selected based on previous toxicological studies that reported neurobehavioral and oxidative effects in zebrafish at similar or higher exposure levels [18,19]. Although direct quantification of SiO_2_NPs in the brain or whole-body homogenates was not performed in our study, previous research demonstrated that these nanoparticles can enter the brain via systemic or intranasal routes and accumulate in neuronal tissue, inducing OS and inflammatory responses [30]. These findings support the relevance of the concentrations used and the observed behavioral and biochemical effects.

Silica nanoparticles and their derivatives (<100 nm) are widely used in biomedical applications and food manufacturing, with global consumption exceeding 1 million tons annually due to their inert properties [31]. Silica nanoparticles have raised concerns regarding their potential neurotoxic effects due to their ability to cross biological barriers, including the blood–brain barrier. Experimental studies indicate that SiO_2_NPs can induce OS, neuroinflammation, mitochondrial dysfunction, and disruption of cellular homeostasis, potentially leading to neuronal damage and altered behavior. The extent of these effects depends on particle size, surface characteristics, dosage, and the developmental stage or health status of the organism. Understanding these mechanisms is crucial for assessing the safety of SiO_2_NPs in both biomedical and environmental contexts [32].

During the social behavior test, although changes in total distance traveled and velocity were not statistically significant, the increasing trend observed in the treated groups, especially at the concentration of 500 μg mL^−1^, indicates a potential stimulatory effect on locomotor activity. This could reflect an increase in exploratory drive, potentially mediated by neurochemical changes, such as upregulation of dopaminergic or serotonergic activity, as previously reported in zebrafish exposed to environmental stressors or neuroactive substances [33]. However, such increased activity could also indicate increased arousal or stress, rather than positive stimulation.

Although these changes did not reach statistical significance, we considered them important to report and discuss as they may reflect subtle early effects of SiO_2_NPs exposure. Presenting these trends contributes to a more comprehensive understanding of the behavioral profile and may guide future studies using larger samples or longer exposure durations.

The reduction in time spent in the stimulus arm, concomitant with increased presence in the no-stimulus arm, implies a decrease in social preference with increasing SiO_2_NPs concentration [34]. This aligns with previous findings that report SiO_2_NPs exposure can interfere with social recognition and affiliative behavior, possibly by disrupting sensory input or central nervous system processing [35].

In the color preference test, a significant decrease in total distance traveled at the highest concentration was observed, along with an increase in swimming velocity [36]. This divergence suggests a shift in locomotor strategy, with shorter distances traveled at higher speeds, which may reflect anxious or erratic movement, commonly associated with environmental stress [37]. Furthermore, the increased preference for the left arm (red) and reduced time in the right arm (green) may indicate that exposure to SiO_2_NPs affects not only exploration, but also chromatic perception or preference. This is relevant, as changes in the visual system have been linked to exposure to metals and SiO_2_NPs in aquatic species [38].

Other behavioral changes, such as increased immobility and decreased mobility at high doses, reinforce the hypothesis of a suppressive effect on spontaneous motor activity [39]. These effects may represent fatigue, neurotoxicity, or a generalized stress response. In addition, the significant increase in both clockwise and counterclockwise rotations at higher concentrations could indicate disorientation, sensorimotor dysfunction, or stereotypic behavior, phenomena that have also been documented in other neurobehavioral toxicity studies.

From a biochemical perspective, the activities of the enzymes CAT, SOD, and GPx reveal a dose-dependent modulation of OS response [40]. The significant upregulation of all three enzymes at 100 μg mL^−1^ suggests an adaptive antioxidant response to moderate oxidative challenge. This upregulation is consistent with the literature describing increased enzymatic activity as a compensatory mechanism to counteract elevated ROS production in the early stages of exposure to SiO_2_NPs [41].

However, at the higher dose (500 μg mL^−1^), a biphasic response is evident: while CAT remains elevated, SOD and GPx activities are slightly lower than at 100 μg mL^−1^, but remain higher than control, indicating a peak adaptive response at the lower dose. This pattern may indicate a threshold of oxidative defense capacity, beyond which the antioxidant system is partially overwhelmed or inhibited [42]. The decrease in GPx and SOD could signal either enzyme depletion or direct interference by SiO_2_NPs with their synthesis or activity.

Although both CAT and GPx use hydrogen peroxide as a substrate, their kinetic properties, cellular localization, and cofactor requirements differ [43]. CAT, mainly in peroxisomes, rapidly decomposes large amounts of H_2_O_2_, while GPx, located in cytosol and mitochondria, requires GSH [44]. At high SiO_2_NP exposure, GSH depletion or direct inhibition may reduce GPx activity, whereas CAT remains active, explaining the differential enzyme activity pattern observed [45].

Similar inhibitory effects at high doses have been reported for other nanomaterials and are associated with oxidative damage, apoptosis, and altered mitochondrial function [46].

Taken together, these results support the hypothesis that SiO_2_NPs exert dose-dependent effects on both neurobehavioral functions and oxidative homeostasis in zebrafish. Moderate exposure (100 μg mL^−1^) appears to trigger adaptive mechanisms, both behavioral and biochemical, while higher exposure (500 μg mL^−1^) leads to behavioral disturbances and suppression of the antioxidant system.

The dose- and size-dependent internalization of silica nanoparticles has been previously documented in vitro, with localization in the cytoplasm, vacuoles, and endolysosomes, which may contribute to OS and cellular dysfunction [47]. Such findings support our observations of dose-dependent behavioral and biochemical alterations in zebrafish exposed to SiO_2_NPs.

The generation of ROS in zebrafish exposed to SiO_2_NPs is closely linked to their cellular uptake. Due to their small size, SiO_2_NPs can enter cells via endocytosis and localize in cytoplasmic compartments such as endosomes, lysosomes, and mitochondria [48]. Interaction with mitochondria and other organelles promotes the production of ROS, including superoxide radicals, hydrogen peroxide, and hydroxyl radicals [49]. This OS triggers activation of antioxidant defense enzymes such as SOD, GPx, and CAT [46]. At moderate exposure (100 μg mL^−1^), this adaptive response is maximized, while at higher concentrations (500 μg mL^−1^), the antioxidant system may be partially overwhelmed, explaining the observed biphasic enzyme activity pattern [50]. Such findings support our observations of dose-dependent behavioral and biochemical alterations in zebrafish exposed to SiO_2_NPs.

The observed behavioral alterations, especially in social preference, mobility, and rotational behavior, are likely interconnected with OS, suggesting that neurotoxicity may be a key mode of action for SiO_2_NPs [51]. Given the widespread use of silica-based nanomaterials in industrial and biomedical applications, these findings highlight the importance of further investigating their potential sublethal effects on aquatic organisms and developing clear concentration thresholds for environmental safety.

The main role of PCNA is to maintain DNA integrity in the event of various aggressions, including oxidative damage. Replication and repair are necessary for DNA integrity, as DNA is frequently damaged by endogenous and environmental toxicants [52]. Under pathological conditions, numerous mechanisms are involved in DNA repair to protect it from damage [53]. PCNA is an essential protein in DNA replication and its function was initially described as an auxiliary protein of DNA polymerases [54,55]. However, PCNA has been shown to affect several vital cellular processes, including chromatin remodeling, DNA repair and cell cycle control [56,57]. PCNA has no intrinsic enzymatic activity and its complex role in cells depends on its ability to regulate other proteins. PCNA interacts with a wide range of enzymes and regulatory proteins, such as cyclin-dependent kinases (CDKs) [57] or the CDK inhibitor p21/waf1 [58], which allows this protein to modulate a wide range of biological functions. In addition, PCNA plays a crucial role in DNA repair under OS conditions [59,60]. Present study found that SiO_2_NPs, an inducer of parkinsonian-like symptoms in humans and primates, induced an increase in the number of PCNA, GFAP, and S100-positive cells in the telencephalon and optic tectum. Another important finding was represented by small areas of necrosis of granular neurons in the PZG area of the optic tectum and areas of neuropil erosion in area central zone III of the TeO white matter. These changes support the involvement of OS caused by SiO_2_NPs in the zebrafish nervous system. It is important to note that exposure to SiO_2_NPs caused activation of cells in the regenerative nuclei of both the telencephalon and the optic tectum.

Although PCNA is primarily a marker of cell proliferation rather than inflammation, its increased expression may also be indirectly linked to inflammatory responses. Changes in PCNA levels can be interpreted in conjunction with other markers such as GFAP, which we also analyzed in this study. In zebrafish, inflammation often triggers proliferative activity during both regenerative and degenerative processes [32]. SiO_2_NP exposure has been reported to induce neuroinflammatory responses through OS, microglial activation, and cytokine release [47,60]. For instance, SiNP-treated microglia showed increased release of TNF-α, IL-1, and IL-6, consistent with an M1-type activation, a hallmark of neuroinflammation.

These findings suggest that the elevated PCNA expression observed in our study could reflect not only regenerative repair but also inflammation-associated proliferative responses. This interpretation is further supported by the upregulation of GFAP and S100 in glial cells, which are known indicators of inflammatory activation in zebrafish [61,62].

In zebrafish, the main neurogenic niches studied in adulthood are located in the telencephalon, optic tectum and cerebellum. The telencephalon remains, without a doubt, the most investigated brain region, as it shares many features and homologues with the mammalian telencephalon, especially when considering adult neurogenesis [63,64,65,66]. In the telencephalon, several studies have explored the identity and diversity of neuronal/progenitor cells that support neurogenic activity observed in different telencephalic subdomains of the zebrafish brain [67,68]. Adolf et al., 2006 [69], through BrdU incorporation and immunohistochemical studies using PCNA, showed that the telencephalon contains two different types of neuronal progenitors: (1) slow-cycling ones, distributed along the ventricular surface, and (2) fast-cycling ones, organized mainly in a subpallial cluster. Slow-cycling progenitors have been identified as radial glial cells (RGCs), whereas fast-cycling cells have been described as neuroblasts [70,71].

Under homeostatic conditions, most neuronal stem cells (NSCs) are considered to be quiescent (resting) type I RGCs. This was observed in the control group. Only a small proportion of NSCs proliferate and express proliferation markers such as PCNA. The latter, so-called type II RGCs, can give rise to committed neuronal progenitors corresponding to neuroblasts (type III cells) [72,73]. When the telencephalon is damaged or subjected to aggression, more NSCs are activated, enter the cell cycle, and begin to express proliferation markers [62], a situation also observed in this experiment in experimental groups. At the same time, NSCs generate an increased number of new neurons compared to homeostatic conditions [74]. Newborn neuronal precursors migrate from the ventricular layer to the site of injury to replace the lost neurons. This regenerative neurogenesis can also be initiated by inflammatory signals [75,76,77].

When the telencephalon is damaged, NSC proliferation is temporarily boosted above the baseline of constitutive neurogenesis, reaching a peak five to seven days after injury [62,72,78]. Notably, lesions inflicted on one hemisphere of the telencephalon result in a proliferative response of NSCs only in the damaged hemisphere, whereas no response is observed in the stem cell niche in the uninjured half of the telencephalon [62,79]. Thus, the signals that trigger stem cell proliferation in response to injury remain confined to the damaged hemisphere. The ventricular zone of the adult zebrafish telencephalon is densely populated by the cell bodies of RG cells, the NSCs of the zebrafish telencephalon [73,80]. RGs express typical NSC markers such as GFAP, brain lipid-binding protein (Blbp) and S100β [63,72,73,81,82]. In all LE, intense expression of GFAP and S100 was observed in the ventricular zone of the telencephalon and mesencephalon, which is densely populated with RG cell bodies, except in the control group. Another experiment shows that the number of proliferating NSCs (PCNA+/S100β+ cells) is reduced in the telencephalon exposed to heat shock in the Tg (hs:bmp2b) zebrafish line, compared to the control group. Furthermore, in vivo cerebroventricular microinjection of morpholine resulted in NSC proliferation [79,83].

The role of S100 proteins in the nervous system of adult zebrafish is not fully understood. Extracellular S100 proteins can regulate apoptosis, proliferation, differentiation, and migration of various cell types: monocytes, macrophages, neutrophils, lymphocytes, myoblasts, epithelial cells, endothelial cells, smooth muscle cells, neurons, and fibroblasts. In addition to serving as calcium-binding proteins, S100 proteins were later discovered as DAMP molecules [84], considered to be intracellular molecules linked to cell death and tissue damage, by inducing a rapid inflammatory response or producing biologically active molecules [85].

They participate in the regulation of intracellular calcium homeostasis, acting as trigger or activation proteins [86] and regulate cytoskeletal stability [87]. However, the functions of S100 proteins in vivo are largely uncertain, as no major changes have been observed in transgenic cells or in transgenic animal models for S100 proteins [88,89]. In addition, S100β protein is produced, stored, and released by astrocytes, tanycytes, oligodendrocytes and radial cells and exerts paracrine and autocrine effects on neurons and glial cells. Thus, it can be assumed that S100 protein participates in the biology of cells that express it. In this way, the expression of S100β defines a late stage of development, and when they express GFAP, they lose their neural stem cell potential [90]. S100 protein continues to be expressed in these regions of adult zebrafish. Glial cells in the subventricular zone play an important role in neurogenesis in adult zebrafish. This explains the presence of GFAP and S100 expression in the subventricular zone in fish of all ages. S100 protein may be maintained in this function in zebrafish throughout their life [91,92].

The expression of the S100 marker increased in both groups exposed to SiO_2_NPs, with a more pronounced effect at 500 mg L^−1^ compared to 100 mg L^−1^. Wei et al., 2020 [93], demonstrated the neurotoxic effect of SiO_2_NPs on adult zebrafish at low (15 nM and 50 nM) and high (300 mg mL^−1^ and 1000 mg mL^−1^) concentrations through behavioral and physiological assays. At low doses (12 ng nL^−1^ of SiO_2_NPs), the number of apoptotic cells in the brain and central nervous system of zebrafish embryos was increased in the presence of SiO_2_NPs, but the difference did not reach statistical significance. Kim et al., 2015 [94] observed that SiO_2_NPs with a diameter of 60 nm diameter affected cells differently than nanoparticles of other sizes, due to enhanced uptake.

Another study found that SiO_2_NPs can cause neurotoxicity by initiating inflammation. Li et al., 2020 [95] found that SiO_2_NPs inhibited memory capacity in adult zebrafish. It has been documented that SiO_2_NPs at concentrations as low as 10 mg mL^−1^ can cause changes in cell morphology, induce OS and apoptosis in both mouse neuro2a and human neuroblastoma cells [96]. Lee et al., 2020 [97] also reported that SiO_2_NPs lead to caspase-dependent apoptosis via endoplasmic reticulum stress in neuronal cells.

A limitation of the current study is the absence of a positive control group using a known neurotoxicant (e.g., ethanol or caffeine), which could have served as a comparative reference point for interpreting the behavioral and biochemical effects of SiO_2_NPs. Future investigations will aim to include such a group to enhance the mechanistic understanding and translational relevance of the observed results.

## 5. Conclusions

The present study provides evidence that exposure to SiO_2_NPs induces dose-dependent effects on both behavioral and OS responses in adult zebrafish. Behavioral assessments revealed subtle but consistent changes, including increased locomotor activity, reduced social preference, disruption of color preference, and increased rotational behavior, especially at the highest concentration tested (500 μg mL^−1^). These changes may indicate neurobehavioral disorders potentially related to stress or neurotoxicity induced by SiO_2_NPs.

Biochemically, moderate exposure (100 μg mL^−1^) triggered an upregulation of key antioxidant enzymes (CAT, SOD, GPx), suggesting the activation of compensatory defense mechanisms against OS. However, at higher exposure levels, a significant decline in SOD and GPx activities was observed, along with a sustained CAT activity, indicating a potential impairment or depletion of the antioxidant system.

Exposure to the two concentrations of SiO_2_NPs tested causes areas of lysis in telencephalon and optical tectum, as well as in the neuropils of the white matter, and the extent of damage increases in a dose-dependent manner The increase in PCNA cells is thought to be due to the activation of more areas of neurogenesis than in mammals. The activation of glial cells, with S100β and GFAP as markers, is similar to the reactive responses observed in mammals; however, it should be noted that such changes may not exclusively indicate neurotoxicity, as they can also occur during normal neurogenesis.

Overall, these findings highlight the dual nature of the biological response to SiO_2_NPs: adaptive at lower concentrations, but potentially negative at higher doses. The observed behavioral changes, in conjunction with the modulation of OS, highlight the need for further investigation into the mechanisms underlying the toxicity of SiO_2_NPs and the establishment of environmentally relevant safety thresholds.

## Data Availability

Data are contained within the article.

## Supplementary Materials

The following supporting information can be downloaded at: https://www.mdpi.com/article/10.3390/life15111715/s1, Table S1. Number of cells positively marked for PCNA, GFAP and S100, after exposure at SiO_2_NP_S_.
