# Supplementary material for: Silicon Dioxide Nanoparticles Alter Social Behavior, Color Preference, Oxidative Stress Markers, and Histological Structure of Brain Regions in Zebrafish (Danio rerio)
Source: Life (Basel). 2025 Nov 5;15(11):1715. doi: 10.3390/life15111715 (PMC12653864; doi:10.3390/life15111715)
Supplement: Supplementary file 1 [file life-15-01715-s001.zip › life-3913112-supplementary.pdf]

**Tectum opticum**	Control	100 µg mL^{-1}	500 µg mL^{-1}
PCNA	56 ± 3.54	153 ± 7.91	238 ± 15.51
GFAP	121 ± 19.04	146 ± 18.11	182 ± 21.55
S100	115 ± 17.10	154 ± 17.68	172 ± 15.81
**Diencephalon**	**Control**	**100 µg mL^{-1}**	**500 µg mL^{-1}**
PCNA	62 ± 13.93	158 ± 18.56	152 ± 15.52
GFAP	64 ± 14.87	188 ± 11.70	256 ± 17.26
S100 beta	60 ± 13.66	129 ± 15.18	234 ± 17.10

Table S1. Number of cells positively marked for PCNA, GFAP and S100, after exposure at SiO_2NP_S
